# The concept of double bundle ACL simulation with a single bundle patellar tendon graft. A cadaveric feasibility study

**DOI:** 10.1186/1758-2555-4-19

**Published:** 2012-06-07

**Authors:** Matthias Jacobi, Robert A Magnussen, Vincent Villa, Guillaume Demey, Philippe Neyret

**Affiliations:** 1 Orthopädie am Rosenberg, Rorschacherstrasse 150, St. Gallen, 9006, Switzerland; 2 Hopital de la Croix Rousse, Centre Albert Trillat, service orthopédie, pavillon R, Groupement Hospitalier Lyon Nord, 103, Grande Rue de la Croix Rousse, Lyon, 69004, France; 3 Department of Orthopaedic Surgery, The Ohio State University School of Medicine, Columbus, OH, USA

## Abstract

**Background:**

There is significant interest in the restoration of the double-bundle anatomy of the native ACL when performing ACL reconstruction. Possible techniques include those utilizing two separate grafts with independent tunnels and those that attempt to mimic this anatomy with a single graft and fewer tunnels. Many of the latter techniques require specific instrumentation and are technically challenging. We demonstrate that the double-bundle anatomy of the native ACL can theoretically be mimicked by a single-bundle reconstruction.

**Methods:**

We performed single bundle ACL reconstruction with a bone-patellar tendon-bone (BTB) graft in two cadaveric knees. Both grafts were placed to mimic the native ACL footprints – one reconstruction was performed with rectangular bone blocks and oval tunnels and one was performed utilizing a standard BTB graft and round tunnels. Qualitative assessment of graft behavior was made as the knees were taken through a range of motion.

**Results:**

The ACL graft was able to qualitatively mimic the behavior of the native ACL in both knees provided the bone blocks were correctly orientated.

**Conclusions:**

ACL reconstruction with a single BTB graft can qualitatively mimic the behavior of the two bundles of the native ACL. The key to ensuring this behavior was noted to be appropriate orientation of the graft in the tunnels. Quantitative biomechanical investigations are necessary to evaluate the impact of graft orientation on function.

## Background

Improvements in understanding of the anatomy and function of the native anterior cruciate ligament (ACL) have lead to increasing interest in reconstructing both bundles of the ACL [1-6]. Different techniques utilizing a variety of grafts, tibial and femoral tunnel configurations, and fixation methods have been proposed [7-11]. Some biomechanical studies have demonstrated increased overall stability following double-bundle reconstruction relative to a single-bundle technique, while others have not [12-17]. Further, while some clinical studies have demonstrated improved stability measurements with double-bundle techniques, improved clinical outcomes have not been consistently demonstrated [10,16,18-20].

Double-bundle reconstruction techniques are not without drawbacks. Many described techniques require the surgeon to prepare three or four different tunnels, requiring the surgeon to place tunnels very precisely [8-11]. The steep learning curve can lead to complications and potentially increase operative time [21]. Because surgeons performing less than 50 reconstructions per year perform the vast majority of ACL reconstructions, development of a simplified technique for creating a double-bundle-like construct would be advantageous [22].

While a technique that mimics double bundle anatomy with a single bone-patellar tendon-bone graft has been previously described, this method requires creation of rectangular tunnels, requiring significant surgical expertise and specific instrumentation [23,24]. We demonstrate that the double-bundle anatomy of the native ACL can be mimicked by a single-bundle reconstruction utilizing rectangular bone-patellar tendon-bone grafts and oval tunnels or with standard grafts and tunnels.

## Methods

### Specimen preparation

Two fresh cadaveric knees were used for this feasibility study, including the knee of an 82 year-old female (knee A) and a 78 year-old male (knee B). Neither knee had undergone prior surgery and both were noted to have normal ACL’s. Some mild degenerative changes of the patellofemoral joint were present in knee A, while the other knee was free of osteoarthritis. To obtain an optimal visualization of the native ACL, the femur, tibia and fibula were cut above and below the knee though their respective diaphyses. Surrounding soft tissues were then removed, leaving only the menisci and cruciate and collateral ligaments.

The medial half of the distal femur was removed with a sagittal saw cut along with the medial collateral ligament, posterior cruciate ligament, and medial meniscus. The anteromedial (AM) and posterolateral (PL) bundles of the ACL were identified and separated. For improved visualization, the AM bundle was colored in red and the PL bundle in blue. This preparation allowed a clear medial view of the native ACL and its two bundles during full range of motion. Following photographic documentation of the native ligament and the orientation of the two bundles in flexion and extension, the bundles were resected and their femoral and tibial attachment sites were marked with the respective color of each bundle.

### Graft harvest and preparation

**Figure 1:**
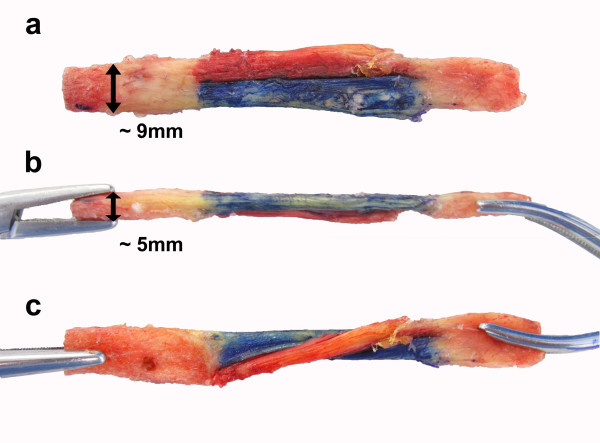
**Single bundle graft.** The graft utilized in Knee A was created with rectangular tibial and patellar bone blocks. **a**) An anterior view demonstrates that the graft has been divided with a longitudinal incision. The portion of the graft that will represent the anteromedial bundle has been colored red and the portion that will represent the posterolateral bundle has been colored blue. **b**) A lateral view of the graft demonstrates that the bone blocks are as this as the patellar tendon in this dimension. **c**) Rotation of the graft demonstrating its two portions.

Before the knee was dissected as detailed above, a bone-patellar tendon-bone graft was harvested from the central aspect of the patellar tendon. For knee A, the bone blocks were rectangular in shape (Figure 1a). From the lateral view, the thickness of the bone blocks was identical to that of the patellar tendon (Figure 1b). For better visualization a longitudinal cut was performed to separate the tendon into two separate parts, which were colored in red and blue (Figure 1a-c). For knee B, a conventional graft was harvested and sized to fit into 10 mm bone tunnels. The graft was divided with a longitudinal cut and colored in an identical manner to that described for knee A.

### Reconstruction

**Figure 2:**
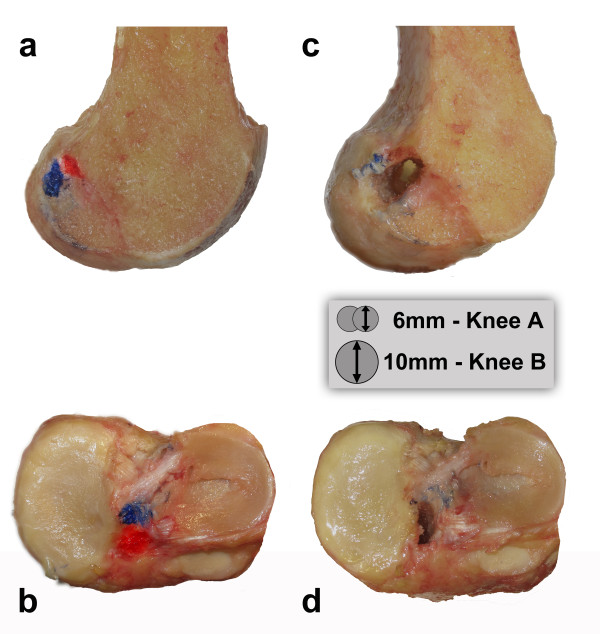
**ACL footprint and tunnel position. a**) A medial view of the femur of knee A following resection of the native ACL. The femoral origins of the anteromedial bundle (red) and the posterolateral bundle (blue) are marked. **b**) A superior view of the tibia of knee A following resection of the native ACL. The tibial insertions of the anteromedial bundle (red) and the posterolateral bundle (blue) are marked. **c**) and **d**) Oval shaped bone tunnels are drilled in both the femur and tibia based on the marked native attachment sites of the ACL. The schematic drawing shows the sizing of the tunnels (knee B isn't shown).

In knee A, two parallel 6 mm tunnels were drilled in both the femur and tibia based on the marked native attachment sites of the ACL. Due to the proximity of the two tunnels to each other, they merged and resulted in oval tunnels on the femur and tibia centered over the native ACL attachment sites (Figure 2). The prepared graft was inserted into the tunnels with the red part representing the AM bundle and the blue part the PL bundle. The graft was fixated for this feasibility study by simple trans-osseous sutures.

In knee B, conventional 10 mm bone tunnels were drilled in the center of the ACL footprints on the femur and tibia and the graft was then placed into the tunnel. The graft was oriented similarly to that in knee A, with the red part oriented toward the anteromedial portion of the footprint on the femur and tibia and the blue part oriented toward the posterolateral bundle footprints. Fixation was achieved by a press fit technique, preventing rotation of the bone blocks in the tunnels.

### Observation of graft behavior

The orientation of the grafts in both knees were photographically documented as the knees were brought through a range of motion from the extended to flexed position. Photographs of the graft position in the fully extended and fully flexed positions were then compared to identify qualitative differences between the native and reconstructed knees.

## Results

### Behavior of the native ACL

**Figure 3:**
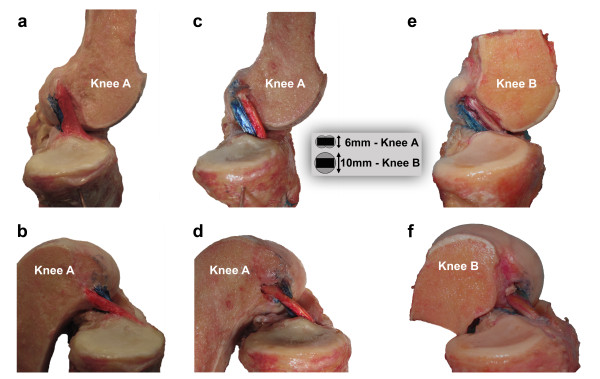
**Physiologic and reconstructed ACL movement pattern.** A medial view of the native ACL of knee A in full extension (**a**) and flexion (**b**). The two bundles are roughly parallel in extension and cross in flexion. A medial view of the same knee following ACL reconstruction utilizing oval drill holes and rectangular bone blocks in full extension (**c**) and flexion (**d**). The graft behavior is qualitatively the same as the native ligament. A medial view of knee B following reconstruction using standard bone blocks and tunnels is shown in full extension (**e**) and flexion (**f**). Again the graft behavior is qualitatively the same as the native ligament. The schematic drawing shows the relation between bone blocks and tunnels in knee A and B.

The femoral insertion of the AM bundle was proximal to the PL insertion on the femur. On the tibia, the AM inserted anterior to the PL bundle. In full extension, the bundles were nearly parallel (Figure 3A). Following flexion of the knee to 120 degrees, the AM bundle was noted to cross over the PL bundle, which maintained a more vertical orientation (Figure 3B).

### Behavior of the reconstructed ACL’s

In knee A as in the native ACL, the two bundles were observed to be parallel in extension (Figure 3C). At 120° of knee flexion the red portion of the graft (representing the AM bundle) was noted to cross over the blue portion of the graft (representing the PL bundle), which maintained a relatively vertical orientation (Figure 3D). The same pattern was noted in knee B in both flexion (Figure 3E) and extension (Figure 3F). The behavior in both reconstructed knees was qualitatively quite similar to that observed in the native ACL.

## Discussion

The main finding of this cadaver feasibility study was that a single bundle patellar tendon graft can mimic the anatomy of the two ACL bundles, provided that it is oriented such that the fibers leaving the anteromedial portion of the ACL tibial footprint and enter the femur near the native femoral origin site of the AM bundle. It was demonstrated that such behavior could be produced not only through the use of oval tunnels, but also through the use of conventional round tunnels.

There are several theoretical advantages to reconstructing the ACL with one graft. First the necessity to use four bone tunnels and four independent fixation devices is avoided, reducing cost and potentially operative time. Second, significant bone loss can occur due to tunnel enlargement following double bundle ACL reconstruction, particularly if tunnel convergence occurs [25]. Revision can be quite difficult in these cases, often requiring two stages. Third, this technique is possible even in knees with a small native ACL footprint in which double bundle reconstruction may be contra-indicated [26]. Potential shortcoming of this technique must also be considered. First, independent tensioning of the different bundles as is performed in double bundle reconstructions is not possible with the described technique. The importance of this limitation should be investigated in a biomechanical study.

The concept of mimicking the double bundle anatomy of the native ACL using one graft placed through a single tibial and femoral tunnel is not new. Several authors have described fixation techniques that allow soft tissue grafts to be used in this manner [27,28]. Shino et al. previously have previously evaluated a similar technique to mimic double bundle function with a single BTB graft [23,24]. They demonstrated that such reconstructions were technically possible in clinical setting and emphasized appropriate graft orientation as in the current study. They did encounter complications while attempting to create rectangular tunnels in the femur including posterior wall blowout, emphasizing the difficulty of this technique.

This cadaveric feasibility study has several weaknesses. First, the focus of the study is to demonstrate qualitatively the behavior of the ACL graft. No attempts were made to quantitatively assess behavior by assessing tension in the different parts of the graft. In addition, the reconstruction was performed in an open manner without utilizing standard arthroscopic instrumentation. The feasibility of performing such a reconstruction *in vivo* was thus not assessed. Further, the removal of soft tissue from the single specimen precluded the performance of any useful mechanical testing of the reconstructed knee to determine the effect of graft orientation on stability.

## Conclusions

A single bundle patellar tendon ACL reconstruction can mimic qualitatively the behavior of the two bundles of the native ACL if the graft is correctly positioned relative to the ACL footprint on the femur and tibia. Quantitative biomechanical investigations are necessary to evaluate the impact of graft orientation on function.

## Abbreviations

ACL: Anterior cruciate ligament; BTB: Bone-Tendon-Bone (patellar tendon autograft); AM: anteromedial; PL: posterolateral.

## Competing interests

The authors declare that they have no competing interests.

## Authors’ contributions

MJ & RAM wrote the manuscript. MJ, RAM and VV performed the study. GD & PN edited and proofread the manuscript. All authors have read and approved the final manuscript.
